# A Survey of Robotic Monocular Pose Estimation

**DOI:** 10.3390/s25051519

**Published:** 2025-02-28

**Authors:** Kun Zhang, Guozheng Song, Qinglin Ai

**Affiliations:** 1 College of Mechanical Engineering, Zhejiang University of Technology, Hangzhou 310014, China; 2 Key Laboratory of Special Purpose Equipment and Advanced Manufacturing Technology, Ministry of Education & Zhejiang Province, Hangzhou 310014, China

**Keywords:** robotic monocular pose estimation, monocular SLAM, single-view OPE, neural methods, robots

## Abstract

Robotic monocular pose estimation is an important part of neural monocular pose estimation-driven methods, which includes monocular simultaneous localization and mapping (SLAM) and single-view object pose estimation (OPE) driven by neural methods. The mapping thread leeches onto robotic monocular pose estimation. Robotic monocular pose estimation consists of the localization part of monocular SLAM and the object pose solving part of single-view OPE. Depth prediction neural networks, semantics, neural implicit representations, and large language models (LLMs) are neural methods that have been important components of neural monocular pose estimation-driven methods. Complete robotic monocular pose estimation is a potential module in real robots. Possible future research directions and applications are discussed.

## 1. Introduction

**Figure 1:**
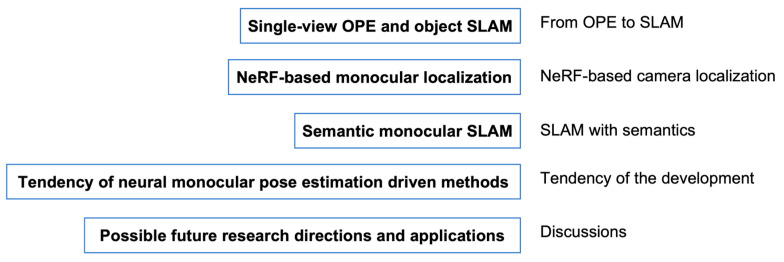
The structure of the survey.

Robotic monocular pose estimation is more comprehensive than monocular pose estimation, which includes camera and object pose estimation. The accuracy of camera pose estimation decides the quality of a 3D map, and the accuracy of object pose estimation decides the fineness of a 3D map. Without reliable and accurate pose estimation, it is impossible to obtain a precise map to require the related applications. Robotic monocular pose estimation methods should be the core of robotic applications that decide the quality of 3D maps. An accurate perception of robots requires accurate robotic monocular pose estimation for robots and objects. Single-view/multi-view OPE mainly focuses on object pose solving and has a relation with monocular SLAM, as illustrated in Section 2.2. Single-view OPE and monocular SLAM are driven by neural methods, including depth prediction neural network, semantics, neural implicit representation, and LLMs [1]. The structure of the survey is shown in Figure 1.

Robotic monocular pose estimation is related to monocular SLAM and single-view OPE. The research on SLAM focuses on jointly solving the task of tracking the position and pose of a sensor and creating a three-dimensional map as it explores an unknown environment. This research field intersects broad topics depending on the sensors used, the given environment with different scales or light conditions, and the required performance, among other factors. Single-view OPE is a widely used technical part of robotic grasping. The 6D pose of objects in the scene should be inferred first to implement the robotic grasping task. The limited mapping part is discussed, which is decided by robotic monocular pose estimation.

The poses of cameras can be defined using the Lie Group of the Special Euclidean Group, while rotation can be defined using the Lie Group of the Special Orthogonal Group [2]. The tangent space for certain elements in the Lie Group of the Special Euclidean (SE) are shown in Lie Algebra [3]. The position of features belongs to the submanifold [4], which decides translation. The clarity of definition of rotation and translation is helpful for optimization. New positions are derived from rotation and translation relative to the original position. The partial derivatives of all points relative to the point interested are used to adjust rotation and translation. Meanwhile, the second-order derivatives are necessary when implementing second-order approximation [5]. Generally, rotation and translation are two separate parts of poses that have different performances.

Traditional monocular SLAM depends on the features detected or the process optical flow goes through to build correspondences, which are helpful to achieve a coarse relative pose among frames using geometric solvers. Therefore, they can be split into two classifications, i.e., indirect and direct, depending on the ways to find correspondences. Traditional monocular SLAM mainly consists of five parts: initialization, front-end, back-end, relocalization, and loop closure. The initialization provides a starting pose for the front-end, the front-end provides coarse pose and structure for the back-end, the back-end optimizes those values, the relocalization launches to recover the process when the tracking is lost, and the loop closure launches to modify the drift of the camera and map when a loop is detected. The front-end, which includes correspondence building, coarse pose solving, and coarse structures of the environment, is a task of computer vision and geometry. The back-end is an optimization task with variables like camera poses and structures of the environment, depending on the format of used reconstructed map, with or without the concept of objects in the environment. With the development of neural methods, traditional monocular SLAM is popular to integrate them.

Visual OPE methods consist of single-view and multi-view OPE methods. Single-view OPE utilizes the captured single image to estimate the pose of objects, while multi-view OPE utilizes the captured images from different views of objects to estimate the poses of objects. Visual OPE, as a neural problem, is becoming increasingly prominent nowadays. The object pose has a translation and rotation of 12 elements in total, which can be predicted by neural networks. The input of those neural networks is the cropped images of objects in most cases, which are with different intensity of different sizes. The structure of neural networks varies, but the input and output of those neural networks are kept the same in most cases. Three-dimensional CAD models are necessary for most visual OPE methods, i.e., model-based methods, which can be used to render accurate images of objects from different views. Those models demand manual work in practical engineering. To obtain a more accurate result, refinement is usually required. The preferred solver to obtain a coarse pose for refinement is PnP + RANSEC based on built correspondences. The refinement procedure is based on the results from PnP + RANSEC as coarse poses to be further refined, usually assisted by 3D CAD models of objects. Visual OPE methods include object detection, which provides number, classification, and bounding boxes of objects. The scene of piling objects should be detected by the object detection module to figure out the bounding boxes of each object. With those detected visible parts of objects, the crop operation provides each object image with a fixed scale to be processed by neural networks, which is the actual input of each object for the neural network. Occlusion should be considered a challenge for visual OPE problems, which heavily affects the size of visible parts of objects and decides the performance of pose prediction.

Neural Radiance Field (NeRF) [6] is a 3D reconstruction neural method to store the structure of the environment with Multi-Layer Perception (MLP), which can be used to render a random view of the environment. NeRF is compatible and lightweight, which can be integrated with monocular pose estimation methods to enhance their localization or mapping. NeRF can be applied to monocular SLAM to make localization efficient and mapping rich. It is also an irreplaceable neural method to achieve model-free single-view OPE. Semantics is a neural method that achieves a more consistent localization and interactive mapping for monocular SLAM. Additional semantic constraints achieve consistent localization, and the interactive mapping is helpful for downstream applications in robotics, such as navigation and grasping. Monocular SLAM can be integrated with a single-view OPE to achieve a more accurate object SLAM. Monocular SLAM provides multi-view images of objects, which hints at the integration of monocular SLAM and multi-view OPE. The potential integrated method is termed SLAM-Dunk style method, which enables more accurate object pose estimation when monocular SLAM is on-going.

This survey focuses on robotic monocular pose estimation. Although t are surveys about SLAM with neural implicit representation [7] and visual OPE [8] separately, this survey should be considered as the first survey focusing on robotic monocular pose estimation and related applications.

## 2. Single-View OPE and Object SLAM

### 2.1. Single-View OPE

OPE by cameras is an important task in robotics. For example, to implement the grasping task for an industrial robot, the first thing to do is to estimate the poses of objects in the scene, e.g., accessories of machines. To benchmark the performance of the proposed methods, Hodan et al. [9] propose a benchmark for object pose estimation, i.e., BOP. They provide datasets and evaluation methods for the challenge of BOP. The leaderboard shows the performance of different methods, and researchers can compare them and have a clear sense of the performance of different methods. A method with a higher score on the leaderboard should be recognized as being of the same importance as a relatively new framework with a relatively low score. All datasets and methods provided by BOP benchmark and leaderboard are with room scale, which are helpful for robotic applications. Room scale is usual for robotic applications, such as robotic grasping. As for large scale, it is convenient to estimate the pose of a satellite by a camera installed on the spacecraft [10] while the experiments are kept on related room- and large-scale datasets using the proposed OPE method. The practical deployment of the spacecraft meets difficulties because of the movement of the spacecraft and the satellite. Meanwhile, the classifications or instances should be identified by robots to implement interactive tasks with human beings. Single-view OPE includes object detection, which detects objects to achieve this target, including the classification and number of certain kinds of objects. The longstanding challenge of OPE is occlusions, textureless objects, poor light condition of the scene, and so on. T-LESS [11] is a challenging dataset, whose objects are with minimal textures and heavy occlusions.

Many scholars classify OPE by the number of images to do the task, such as the classification in CosyPose [12]: 1view, 4-views, and 8-views. The setting of a single-view OPE is described as follows. Given a single image captured by a camera with the known pose, the poses for objects in the scene should be inferred from the given image. Multi-view OPE estimates object pose from multiple images from different views. Single-view single-object OPE is a simplified case. There is one detected object in the scene or a split detected object in a cluster scene with or without occlusion. The light condition and the complexity of piling objects which directly affect occlusions are challenging for OPE. Although multi-view methods can handle difficult cases somehow, those methods demand different images captured by different views, which demand a huge cost of labor compared to single-view OPE, although the accuracy is improved in some way. Physically based rendering (PBR) images are synthetic images that can be used for training to obtain a set of rough neural parameters. To be more specific, an OPE neural network can be trained using PBR images and tested on real or synthetic test images. There are several tricks using mixed PBR images and real training images for training to achieve better testing performance than using PBR images only. Training, using PBR images only, is limited because of the problem of domain adaptation. The results from training on PBR images can be utilized as a referring starting point or fast testing point to test whether a neural network architecture is suitable or not among various tasks of research work. When properly solving the problem of domain adaptation, using PBR images only is an economical way to reduce manual work in practical engineering. Object coarse pose in CosyPose is generated by a single-view single-object 6D pose estimation method. Object coarse pose is matched through different images from different viewpoints to jointly estimate camera and object pose. Global scene refinement is achieved by an object-level bundle adjustment to jointly refine the poses of the camera and the object. The refinement procedure is inspired by DeepIM [13], which is a render-and-compare approach. Noted iNeRF [14] can be regarded as a NeRF-based render-and-compare approach to refine camera poses based on observations of an object or scene. Object-level correspondence is used to recover the relative position of the camera. Object-level bundle adjustment is used to improve noisy single-view object poses through in a global refinement procedure. Multi-view consistency is optimized to achieve a single consistent reconstruction of the scene. The multi-view solution significantly improves pose estimation and 6D detection. Coupled iterative refinement [15] refines pose as well as correspondence in a tightly coupled manner based on object models with an RGB or RGB-D input. Bidirectional Depth-Augmented Perspective-n-Point (BD-PnP) is proposed to solve the optimization problem of pose refinement, which is a differentiable layer. RAFT [16] is utilized to construct optical flow between the input image and a set of rendered images of a known 3D object. The flow and pose are updated jointly and conditioned on the current pose or the flow. Pose is updated by a GRU-based update operator. The method consists of three stages, i.e., object detection, pose initialization, and pose refinement. Object detection and pose initialization follow CosyPose. The main contribution should be considered pose refinement. Model-based methods of unseen objects are more challenging than the methods above. GenFlow [17] is an optical flow-based method for 6D pose refinement of novel objects, which can also be utilized for seen objects. An RGB image and 3D models of target objects are unseen during training and the goal is to estimate 6D poses of objects with respect to the camera. Accuracy and generalization are balanced with the help of the target object’s shape. Optical flow is predicted between the rendered image and the observed image to refine 6D pose iteratively. A constraint of 3D shape and the generalizable geometric knowledge from an end-to-end differentiable module are enhanced by the predicted optical flow. Multi-scale correlations and coarse-to-fine refinement improve performance. The pose hypotheses are generated by GMM-based sampling. High-scoring hypotheses are refined with the pose refiner. The refinement consists of feature extraction and GenFlow update module. GenFlow update module consists of GRU and a differentiable PnP submodule. In a word, a scene with clustering objects is detected, and every detected object needs to be estimated, whether they have the same class or not. In addition, occlusion usually happens when objects are piled together. This case brings a huge challenge depending on the visible part of each object. Object detection includes the classes of objects, the number of objects of each class, and the visible shape of each object. The object poses are decided by their visible part.

A cluster of objects, which is a usual scene in BOP datasets, should be separated into individual objects, which can be referred to as instances with or without occlusion. Inspired by neural 3D reconstruction methods, reconstructed 3D models of objects can be generated from captured images, which can be used to render random views. It is promising for practical applications because of less labor and more economic benefits. Noted iNeRF optimizes camera pose, given the initial guess of pose and NeRF of scenes. A proper supervision signal and an iterative method to refine poses are important. The iterative method in iNeRF is gradient descent, and the supervision signals are the intensity differences between two images. Objects can be reconstructed at first using neural implicit representations, such as NeRF, DVGO [18], Plenoxels [19], TensoRF [20], or Instant-NGP [21]. The optimization of camera poses can be implemented based on the corresponding trained neural implicit representation. This inverse operation from trained neural implicit representation to iterative camera pose, which can be used to render every image of every pose, is called iNeRF style refinement. Several methods support differential backward so that corresponding pose refinement can be implemented by corresponding neural implicit representation, e.g., DVGO.

**Figure 2:**
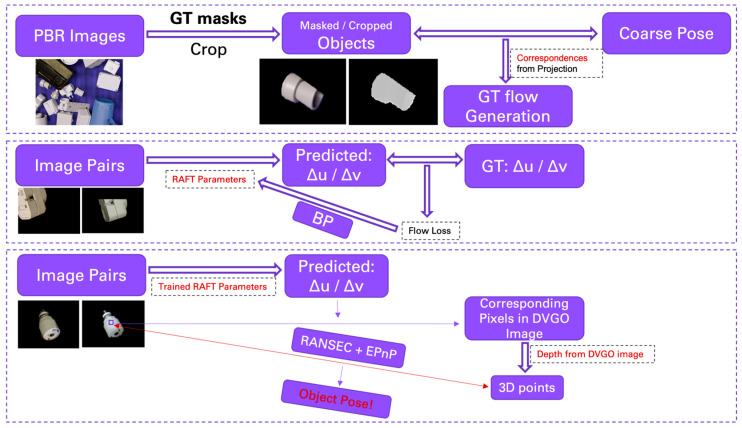
The pipeline of iNeRF-OPE.

**Figure 3:**
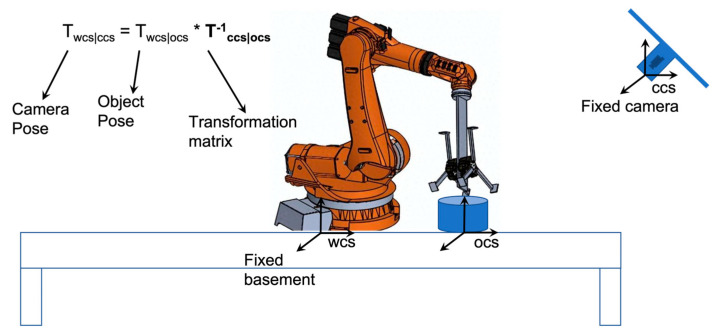
An example of robotic grasping with a single object.

**Figure 4:**
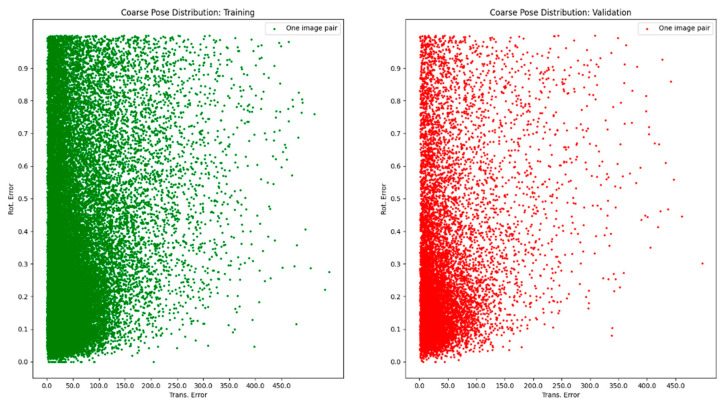
The distribution of coarse pose.

The potential work to integrate inverse operation of neural implicit representation into OPE methods is termed iNeRF-OPE. The pipeline of iNeRF-OPE is shown in Figure 2. The inverse operation of neural implicit representation in iNeRF-OPE is to recover depth information of rendered images from DVGO. The difficulty is induced by transformation and domain adaptation. The transformation is from scene-level to object-level and from camera pose to object pose. A scene with many clustering objects should be transformed into the object level one by one with the help of object detection. The results are a camera pose, which should be transformed into an object pose using the equation illustrated in Figure 3, which is the transformation between the camera coordinate system and object coordinate system. The transformation from object pose to camera pose is similar to the transformation of certain feature points from object frame to camera frame defined in [22]. The distribution of coarse pose is shown in Figure 4, which is very dispersive for objects in the T-LESS dataset, so iNeRF style refinement is invalid and given up. The core of the iNeRF-OPE pipeline is the correspondence built by RAFT, which is an optical flow prediction neural network, and the pose is solved by RANSEC+EPnP. This pipeline meets a domain adaptation problem, which should be handled further. The RAFT is trained on PBR images whose ground truth flow can be generated by geometric projection. While the trained RAFT is applied to real images to obtain the predicted flow, with depth from DVGO, a pixel can be transformed to a 3D point in the object coordinate system. The object poses can be obtained by RANSEC+EPnP solver-given pixels and corresponding 3D points.

The OPE methods on the BOP leaderboard are all model-based. It is a popular way to obtain a relatively high score on the leaderboard. The average score achieved by iNeRF-OPE on T-LESS dataset is around 0.5, which is lower than most model-based methods. The experiments include RGB and RGB-D settings. The depth sensors require additional cost and deployment and have a limited sensing range, which is not suitable for outdoor scenes. The OPE field should not be limited to indoor scenes, which limits the development of the research of real robots. The possible advantages of iNeRF-OPE should be considered due to the following two points. It is a model-free method which reduces labor in practical applications though the separate pipeline demands relatively large investment, which can be improved further possibly; and it is a helpful hint for complete robotic monocular pose estimation, which benefits the robotic community.

Present tasks on BOP benchmark still lack model-free 6D object localization tasks; iNeRF-OPE should be considered the first work implementing the model-free task of object localization, which the current BOP benchmark lacks for one of the BOP datasets, i.e., T-LESS. NeuSurfEmb [23] is a successful model-free method, which is similar to iNeRF-OPE in some ways. The experiments are conducted on the LINEMOD-Occlusion [24] dataset and real world. The real-world experiments benefit the robotics community.

Robotic grasping is an important research topic both in the academic and industrial worlds, and the process of robotic grasping is divided into multidisciplinary research topics. To implement a grasping task for a single object by a robot, the first thing to do is to identify the object pose and then generate a grasp pose for the manipulator [25]. The process of grasping is described as follows. For certain mechanisms of manipulators, the grasping pose is generated with the help of OPE for target objects. The whole grasping process is implemented after route planning. The process is performed by manipulators. The research of robotic grasping includes mechanics, actuators, mechatronics, etc. The process of grasping is similar to very low-speed automatic driving on a manipulator if there are cameras installed on the manipulator. NeRF has practical applications in robotic grasping. Dex-NeRF [26] utilizes NeRF to implement robotic grasping tasks, though the usage is limited. The objects are transparent, and depth sensors are usually unable to obtain their depth information. However, the way to generate a grasping pose is still data-driven. The input includes depth rendered from NeRF, and the output is a grasping pose from Dex-Net [27]. Depth rendering is for all objects in the scene, which is different from the OPE methods. Nevertheless, Dex-NeRF provides an example of the application of NeRF in robotic grasping.

### 2.2. Object SLAM and OPE Plugging

The main differences between object SLAM and traditional SLAM are the additional thread of object detection and the potential thread of OPE. Object detection is not yet mature, as it is in its early stage. Object detection provides monocular SLAM with additional constraints, which can be referred to as camera–object constraints. OPE provides a richer and more accurate representation of the reconstructed map. OPE usually includes object detection. OPE can provide not only labels but, also, additional accurate object poses, which is helpful for object mapping and camera localization.

Salas-Moreno et al. presented SLAM++ [28] in 2013. They do incremental SLAM at the object level with a hand-held depth camera in large, cluttered environments of indoor scenes. They focus on practical applications of augmented reality. The full pipeline includes live frame surface measurement, live camera pose estimation, object insertion, pose update, pose graph optimization, and surface rendering. The full pipeline demands a live depth map. Therefore, the full pipeline is regarded as RGB-D. The live camera poses are tracked with the ICP algorithm. The core contribution of SLAM++ is to introduce a camera–object constraint to the graph optimization. The surface reconstruction part refers to KinectFusion [29], whose setting is also RGB-D. As illustrated in CubeSLAM [30], detected objects can provide geometric and scale constraints to improve the accuracy of ego-motion estimation and reduce drift. Object detection provides bounding boxes for objects.

Exploiting a reliable way to integrate OPE and SLAM is of more value to research communities, although the contents of research are hard and heavy. These two methods are related in some senses. The main difference is the specific target. Both of them focus on the estimation of poses. However, single-view OPE focuses on estimating the pose of an object, while monocular SLAM focuses on evaluating the poses of the camera. PnP + RANSEC is a welcoming solver to do pose estimation. When the target is the camera pose, the chosen 3D points should be in the camera coordinate system. When the target is an object pose, the chosen 3D points should be in an object coordinate system. Two-dimensional pixels are always in the coordinate system of the camera. When the solver is used in practical applications, the coordinate systems for the chosen 3D points are the camera coordinate system or object coordinate system. The pose of the camera or object is the transformation of the world coordinate system and the camera or object coordinate system, including rotation and translation. Bounding boxes provide each object with the object coordinate system. A map, similar to how humans construct it in the brain, will be provided by SLAM-Dunk style methods. This attempt will be possible to achieve in the near future, as demanded by real robots.

## 3. NeRF-Based Monocular Localization

Visualization of the environment in which robots are moving around is important to both robots and human beings. The fast development of neural reconstruction methods boosts the development of monocular SLAM and robotic perception. The rendering process of NeRF is a traditional physical process that has apparent equations to explain. NeRF combines MLP and rendering processes to reconstruct the environment. Rendered images provide cues to align images from different viewpoints, which is helpful for pose estimation. Noted iNeRF handles NeRF-based monocular localization of a scene in some senses with the help of rendered images of each iteration step. It should be considered as a NeRF-based iterative image alignment method. However, this attempt is limited because of the required reasonable starting camera poses, which should be near the ground-truth camera poses. Provided images in monocular SLAM are adjacent, which naturally overpass the problem of reasonable camera poses, where iNeRF style plays an important role. The pose refinement of iNeRF is supervised by the intensity difference between the ground-truth image and the rendered images of each step. The camera pose has a translation vector and rotation matrix with 12 elements in total, while the intensity difference is the photometric difference between two images. To be more specific, given the trained NeRF of a scene, optimization goes well only when starting poses are close to the ground-truth poses, and the optimization process is generally backpropagation. In addition, it is hard to create a novel optimization method to handle local minima. Alternative strategies are chosen to overcome this shortcoming. Engineering attempts are used to handle the pose refinement with reasonable coarse camera poses when initial camera poses are relatively large, i.e., sampling in the space of pose hypothesis and choosing the best pose, which is nearest to the ground-truth pose.

This strategy was proposed by Lin et al. [31], transferring the initial camera pose with a large offset to a coarse camera pose with a small offset. The work of Bundle-Adjustment Neural Radiance Fields (BARF) [32] aims to train NeRF that is given a set of images with imperfect camera poses. In other words, it jointly learns neural representations and registers camera frames. Original NeRF demands accurate camera poses of images to learn the scene representation, which costs labor. BARF is equipped with classical image alignment and coarse-to-fine registration. Original positional encoding has a negative impact on registration. Imperfect poses should be in reasonable spaces. CodeNeRF [33] is similar to NeRF, which is scene-specific. The variation in object shapes and textures across a category can be trained from a set of posed images, which can be used to synthesize novel views of unseen objects. At test time, CodeNeRF jointly optimizes the camera viewpoint, shape, and appearance. Unseen objects can be generated from a single image. NeRF − − [34] jointly optimizes camera intrinsics, camera poses, and a NeRF model. Camera parameters are regarded as learnable parameters while NeRF is training.

A contemporaneous work of iNeRF named iMap [35] is the first method to do RGB-D SLAM using neural implicit representation. A keyframe structure, multi-processing computation flow, and dynamic information-guided pixel sampling comprise the method. Efficient geometry representation and plausible filling-in of unobserved regions can be considered the main advantages compared to traditional dense SLAM. A parallel tracking process continuously optimizes the pose of the latest frame to the fixed scene network. This tracking process is similar to the setting of iNeRF. In the mapping thread, the tracked pose is refined. ONeK-SLAM [36] is a robust NeRF-based object-level SLAM method. It uses information at the object level to improve the method. Object-level pose estimation constructs the objects’ reprojection errors and then minimizes the reprojection error for all objects to receive camera poses. The whole scene is decomposed into individual objects, and each object’s shape and representation are shown as individual NeRF. Based on object-level feature points and NeRF, object-level optimization jointly handles geometric and global characteristics of objects to refine objects’ poses. In addition, for dynamic objects, ONeK-SLAM detects and eliminates them.

NeRF-based localization is a NeRF-based image alignment problem that starts from iNeRF. It opens an era for the robotic and SLAM communities to introduce those neural representations into the localization problem. The iNeRF style is an important choice for NeRF-based monocular SLAM and a potential choice for single-view OPE.

## 4. Semantic Monocular SLAM

### 4.1. Traditional Semantic Monocular SLAM

Semantics rooted in handicrafts or neural methods can be applied to monocular SLAM or 3D reconstruction, which is an assistant for monocular pose estimation-driven methods, especially monocular SLAM. With the insertion of semantics, the mapping part of monocular SLAM is more interactive, and the localization part has more constraints. Semantics can be considered an important neural method to help monocular SLAM, which has a high-level ability to interact with human beings, as illustrated in Section 5. On the one hand, semantics enriches the representations of the environment, which are helpful for the interactions with users; on the other hand, semantics provides more constraints for monocular SLAM. Semantic monocular SLAM is promisingly assisted by NeRF, as discussed in Section 4.2.

Early attempts are based on databases or templates created beforehand because of the limitation of feature engineering, object detection, and semantic segmentation. Traditional semantic monocular SLAM estimates camera parameters, scene points, and object labels using both geometric and semantic attributes in the scene. More elements in the scene are used in further research, i.e., points, regions, and objects to achieve more abundant recovery and robust estimation. Castle et al. [37] utilized planar object recognition from a database built beforehand, therefore with semantics, to enhance the ability of localization and enrich mapping. It is a planar object SLAM with semantics in some sense. Localization of planar objects improves the localization of cameras and enriches the representation of the map. The thoughts from this work are pioneering. Civera et al. [38] propose an algorithm for semantic SLAM that merges traditional meaningless points with known objects. The method is an EKF monocular SLAM with an object detection thread. Bao et al. [39] present a semantic structure from motion (SSFM) method, which could jointly recover camera parameters and scene points as well as locations, poses, and categories of objects in the scene. Dame et al. [40] propose a dense reconstruction method utilizing 3D object shape priors. Given object shapes and coarse poses for objects, the object pose can be estimated by a refinement process. Segmentation enhances the maps’ clarity, accuracy, and completeness. Three-dimensional data helps the segmentation process.

The concept of semantic monocular SLAM was ambiguous in early attempts because of the limitation of computing ability and the applicable traditional pipeline. It becomes clearer, since SLAM++ and Semantic BA [41] are related to semantics and object detection. Semantics is an additional helpful method. The limitations of that era can be concluded as follows: the first point is the computing ability and DL frameworks are limited, the second point is the related research areas, e.g., semantic segmentation, are in their infancy and the third point is the referable works are almost none. Semantic BA can be described in detail as follows. Objects are important to find correspondence. Object detection provides labels and bounding boxes for objects. Monocular SLAM can interact with useful content when the background and objects are identified. Semantic edges are introduced, which refer to frame-to-landmark and frame-to-object constraints. Frame-to-object edges construct reprojection errors by the corresponding reprojection distance of points with labels. Frame-to-frame constraints are introduced into bundle adjustment. Spontaneously, probability is introduced by accounting for the reliability of matching different types of features. Li et al. [42] propose a dense SLAM-based scene understanding method consisting of single-view object classification, semantic segmentation, and object pose estimation. The core idea of this approach integrates a probabilistic inference scheme that predicts semantic labels for object hypotheses at each new frame with incremental dense SLAM. Bowman et al. [43] propose a probabilistic data association for semantic SLAM with relatively high-level representations. Feature and object detections are parallel. They tightly couple inertial, geometric, and semantic observation into a single optimization framework. The optimization method is expectation maximization (EM) [44]. However, probabilistic data association refers to features rather than objects. The early attempts used semantics to help visual SLAM focus on using prior semantic knowledge about geometry to improve reconstructed maps. The maps are constructed by jointly optimizing semantics and geometry. Toft et al. [45] and VSO [46] share the idea of projecting semantically labeled 3D points into semantically segmented images, constructing error maps for each class through distance fields, and then using these errors to refine the camera pose. The former aims to do long-term relocalization, and the latter is designed for visual odometry, optimizing camera poses while constructing a labeled 3D point cloud. SemanticFusion [47] aims to fuse the semantic segmentation from CNN based on ElasticFusion [48] to generate a 3D map with semantic labels. Meanwhile, this framework also leads to an improvement of 2D semantic labeling over frames from CNN predictions. On most occasions, semantic mapping is not only assigning semantic labels to 3D points from semantic segmentation but also an optimization problem improving the results of semantic segmentation prediction.

Scene understanding is related to semantic monocular SLAM in some senses. The results from scene understanding can help monocular SLAM, especially multi-task monocular SLAM. Tosi et al. [49] propose a method for scene understanding from video. They combine depth, motion (optical flow), and semantic predictions to achieve better performance of scene understanding. The three tasks are jointly addressed by a novel training protocol and compact network architecture. MSA R-CNN [50] is a remote sensing scene understanding method that includes a super multi-scale feature extraction network (SMENet), an adaptive dynamic inner lateral (ADIL) connection module, and a distributed lightweight attention module (DLAM). The scenario of remote sensing scene understanding is outdoor with a large scale. Semantics helps monocular SLAM handle dynamic scenes and optimization, which are both challenges for traditional monocular SLAM. DSOD [51] is a monocular odometry based on DSO aiming at handling the dynamic scene with the help of semantic segmentation and depth network. The depth prediction network provides initial depth. Semantics is used to detect potentially moving objects. A movement consistency check decides if a potential moving object is a real moving object. Real dynamic points are outliers, and fake dynamic points are inliers. Bao et al. [52] propose a semantic visual direct odometry. They exploit the direct alignment of semantic probabilities and a joint error function to achieve better convexity, which is different from photometric error. Photometric error induces non-convexity when tracking points with large displacement. The joint error function achieves better convexity. Jacobian matrixes of the residual of semantic channels attend the windowed optimization problem. Semantics has promising applications in collaborative SLAM (CSLAM), which serves multiple robots. Multi S-Graphs [53] aims to serve a multi-robot SLAM in the indoor scene. It utilizes high-level semantic-relational information to generate cooperative maps and localize robots. Semantics provides relational information for the CSLAM. A room-based descriptor is proposed to perform inter-robot loop closure. Most CSLAM methods rely on raw sensor measurement and low-level features, which usually cause wrong loop closure and limit scalability.

In conclusion, scene understanding is related to semantic monocular SLAM, which can be applied to various applications, dynamic scenes are the main challenge for monocular SLAM, which can be handled with semantics, and optimization of monocular SLAM can be improved with the help of semantics.

### 4.2. NeRF-Based Semantic Monocular SLAM

SNI-SLAM [54] is an RGB-D semantic SLAM with neural implicit representation to achieve accurate semantic mapping, surface reconstruction, and camera tracking. A hierarchical semantic representation is introduced for multi-level semantic comprehension. The appearance, geometry, and semantic features are integrated through cross-attention for feature collaboration. Thus, it remains robust when the single attribute is defective. An internal fusion-based decoder and a feature loss updating the scene representation at the feature level, which tends to guide the network optimization better rather than low-level losses, are designed. SGS-SLAM [55] is a dense RGB-D semantic SLAM combined with Gaussian Splatting. Three-dimensional Gaussian splatting is an alternative to NeRF, with fast training and rendering speed [56]. Appearance, geometry, and semantics are optimized through multi-channel. Semantic feature loss is introduced to improve depth and color loss usage in object optimization. Semantics provides supervision for parameter optimization and keyframes selecting. Scene editing and manipulation are supported by its accurate disentangled object representation. SGS-SLAM replies on provided depth and semantics. It demands large memory consumption in large scenes.

## 5. Tendency of Neural Monocular Pose Estimation Driven Methods

**Figure 5:**
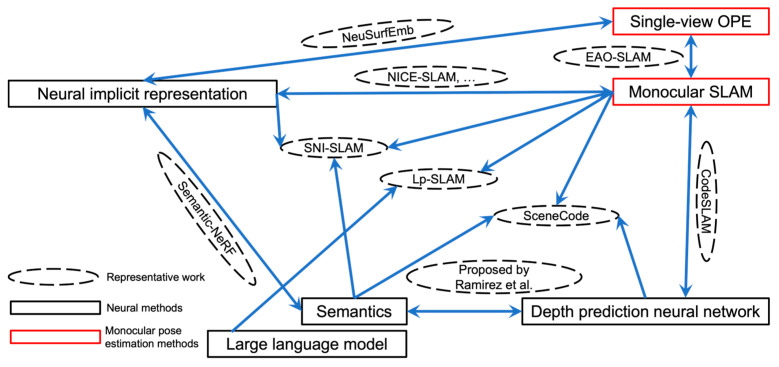
The tendency of neural monocular pose estimation-driven methods and semantic plugging.

The tendency of neural monocular pose estimation-driven methods benefit from the development of neural methods, e.g., depth prediction neural network, semantics, neural implicit representation, and large language model. Those neural methods usually have unique abilities to make the localization more accurate, the built map richer, and more interactive with human beings. The tendency of monocular pose estimation-driven methods is shown in Figure 5. Depth prediction neural networks, semantics, and monocular SLAM could interact with each other to achieve a better performance before neural implicit representation became a popular neural method.

Semantics can help with depth neural networks, which are shown as semantic plugging. Ramirez et al. [57] propose a method to integrate semantics and depth prediction neural networks to enhance the performance of the latter. CodeSLAM [58] utilizes the results of depth prediction from neural networks to enhance the monocular SLAM. SceneCode [59] is a 3D dense reconstruction method with semantics that can jointly estimate motion, geometry, and semantics in a unified optimization following CodeSLAM. NeRF changes the technical distribution of monocular pose estimation. Three attempts can be regarded as having important influences in this technical distribution. The first attempt is to integrate semantics and NeRF, i.e., Semantic-NeRF [60]. It introduces semantics into the NeRF rendering process to obtain rendering results with semantics. The second attempt is to integrate NeRF and monocular SLAM to achieve a complete monocular SLAM, i.e., NICE-SLAM. The third attempt is to incorporate semantics, NeRF, and monocular SLAM, i.e., SNI-SLAM and SGS-SLAM. Single-view OPE can be integrated with neural implicit representation to achieve model-free methods, such as NeuSurfEmb and iNeRF-OPE. Single-view OPE can be integrated with monocular SLAM as well, e.g., EAO-SLAM [61]. EAO-SLAM integrates a single-view OPE method, i.e., iForest, and a monocular SLAM to achieve a richer object SLAM. Wu et al. [62] propose an object SLAM framework for robotic grasping, which can be considered as a successive work of EAO-SLAM. On the side of interactivity with human beings, Lp-SLAM [63] integrates a user interface module with an RGB-D SLAM. The query from users can be transformed into position instructions, which helps the SLAM. The applied neural method is Large Language Model (LLM), and the attempt is in its infancy. Multimodal Large Language Models (MLLMs) have demonstrated an excellent ability to understand images and 3D data. However, MLLMs lack an understanding of the appearance and geometry of objects. Amaduzzi et al. [64] propose the first framework combining NeRF and MLLMs to handle the difficulty of appearance and geometry of objects, which has promising applications in robotics. MLLMs have their limitations as mentioned, while NeRF can handle appearance and geometry.

Regarding SLAM as a pure neural network prediction problem, it is limited. Monocular SLAM requires heavy generalization, which fixed neural networks fail to achieve. Front-end, loop closure, and relocalization can be assisted by neural methods, which are data-driven and have the limitation of generalization in practical applications. The back-end has the potential to integrate with neural methods. Data-driven methods require a large amount of accurate training data, which cost labor and investment. The balance between traditional geometric methods, traditional optimization methods, and neural methods is important while designing a monocular SLAM. Single-view OPE can be integrated with the monocular SLAM and has the potential to integrate several neural implicit representations. Monocular SLAM can be integrated with single-view OPE. The combined method can integrate neural methods such as a depth prediction neural network, semantics, and a neural implicit representation in the future. With present technologies, it is possible but still dififcult. True robots demand the combined method above to become reliable companions for human beings.

## 6. Possible Future Research Directions and Applications

SLAM methods with the interactive ability with human beings, which real robots demand, are termed as intelligent SLAM style methods, such as Lp-SLAM shows, although the attempt is in its infancy. Neural monocular pose estimation driven methods have potential applications in civil engineering. Scan-to-BIM [65] is a traditional way to turn the obtained point cloud data into a Building Information Modeling (BIM) model, and it is not an automated process. Asadi et al. [66] propose an automated registration of a series of image frames to an as-planned BIM in real-time, taking advantage of monocular SLAM. Inversely, camera poses can be improved by BIM. Robots can potentially be deployed with neural monocular pose estimation driven methods and can potentially replace human beings in dangerous working environments. The blueprint of the life of human beings in the future should be considered as living and working with real robots. The promising applications of neural monocular pose estimation-driven methods are shown in Table 1. The tight integration of SLAM and OPE, i.e., SLAM-Dunk style methods, which are possibly the final morphologies of neural monocular pose estimation-driven methods, can possibly be deployed to applications in various research domains, including robotics, mechanical engineering, civil engineering, automatic driving, and real robots.

## 7. Conclusions

Robotic monocular pose estimation is a core technical part of neural monocular pose estimation driven methods and has various applications in robots, automatic vehicles, and VR/AR devices, among others. Depth prediction neural networks have fundamental functions in neural monocular pose estimation-driven methods. Semantics is an important cue for robots to understand the environment and the interaction between robots and human beings. With the development of NeRF, it plays an irreplaceable role in monocular SLAM. LLM can be considered an important neural method to achieve interaction with human beings for neural monocular pose estimation-driven methods. The iNeRF-OPE can be considered an attempt for complete robotic monocular pose estimation, which integrates monocular SLAM and single-view OPE in some senses. The complete robotic monocular pose estimation, i.e., the integration of the localization part of monocular SLAM and the object-solving part of single-view OPE, is a potential module for real robots.

## Figures and Tables

**Table 1:** Multidisciplinary applications of neural monocular pose estimation driven methods.

Research Domain	Subdomain	Promising Methods
Mechanical Engineering	Intelligent Manufacturing	SLAM + OPE
Civil Engineering	Intelligent Construction	SLAM + OPE driven BIM
Automatic Driving	Perception	SLAM + OPE
Real Robots	Real Perception	SLAM + OPE

## Data Availability

No new data were created or analyzed in this study. Data sharing is not applicable to this article.
